# Extent and Effects of Recurrent Shortages of Purified-Protein Derivative Tuberculin Skin Test Antigen Solutions — United States, 2013

**Published:** 2013-12-13

**Authors:** Rebekah E. Turner, Andrew J. Heetderks, Awal D. Khan, Terence L. Chorba, John A. Jereb

**Affiliations:** Div of Tuberculosis Elimination, National Center for HIV/AIDS, Viral Hepatitis, STD, and TB Prevention, CDC

Two purified-protein derivative (PPD) tuberculin skin test (TST) antigen solutions are approved by the U.S. Food and Drug Administration (FDA): Tubersol (Sanofi Pasteur Limited) and Aplisol (JHP Pharmaceuticals, LLC). Tubersol was out of production in late 2012 through April 2013 (1). Shortages of Aplisol have resulted from increased demand as practitioners have sought a substitute for Tubersol. Tubersol production resumed in May 2013, and supplies had been nearly restored by early June. However, in mid-July, state tuberculosis (TB) control officials notified CDC of difficulty obtaining Tubersol and Aplisol. Sanofi Pasteur notified FDA of a temporary delay in the availability of tuberculin in the 10-dose and 50-dose presentations. In mid-October, the 10-dose presentation was being returned to market, on allocation, which means that historical purchasing practices determine the amount that customers are allotted. In late October, the 50-dose presentation was being returned to market, also on allocation, one vial per historical customer per month. Supplies are forecast to approach normal during January 2014, after distributors have restored their supply chains. A compensatory surge in testing after deferment of testing during the periods of shortage might cause further temporary instability of supplies. In mid-August 2013, officials in 29 of 52 U.S. jurisdictions noted a shortage of at least one PPD TST antigen solution in health departments to the extent that it interrupted activities. This report includes a summary of the extent and effects of the shortages and a reiteration of advice on how to adapt to them.

Two kinds of immunologic tests are used for detecting *Mycobacterium tuberculosis* infection: TSTs* and interferon-γ release assay (IGRA) blood tests (2). The indications for using these tests are the same, although one or the other test is preferred for certain populations (e.g., TST is preferred for children aged <5 years) (2). These preferences could play a role in setting priorities when one of the methods is unavailable. Together, these tests are the only means for detecting latent *M. tuberculosis* infection, and they contribute to diagnosing active TB disease. When findings such as chest radiography and mycobacterial cultures are sufficient for confirming or excluding the diagnosis of TB disease, the results from TST or IGRA might be unnecessary. However, most TB cases in the United States are diagnosed with an array of diagnostic findings, including results from TST or IGRA. When TB disease is strongly suspected, specific treatment should be started, regardless of results from these tests.

In mid-August 2013, during their routine assessments of the 68 TB control programs that are funded by federal cooperative agreements, program consultants at CDC’s Division of Tuberculosis Elimination inquired about shortages of PPD TST antigen solutions. The officials in 52 jurisdictions (76%), including 43 states, two unincorporated territories (Puerto Rico and the U.S. Virgin Islands), and seven cities (Houston, Texas; Los Angeles, California; New York, New York; Philadelphia, Pennsylvania; San Francisco, California; San Diego, California, and the District of Columbia) shared updates. These jurisdictions accounted for 93% of the TB cases reported in the United States in 2012 (3). Officials in 29 jurisdictions (56%) reported shortages of PPD TST antigen solutions in health departments (10 Tubersol only, four Aplisol only, and 15 both) to the extent that routine activities were being threatened or had been curtailed. When comparing the shortages in August with those in early June, immediately before Tubersol supplies had become available again, officials in 13 of these 29 jurisdictions described the shortages in health departments as more severe in August; in seven, they described the shortages as similar; in six, they described the shortages as less severe; and in three, they were unsure about the comparison. Officials in 40 jurisdictions (77%) reported that they had been alerted about shortages by health-care providers in a non–public-health sector (19 Tubersol only, one Aplisol only, and 20 both). In four of the 23 jurisdictions that were not experiencing shortages at health departments, some routine testing practices (e.g., screening of matriculating students) were suspended preemptively. Use of IGRAs was being increased in 12 jurisdictions in response to shortages. The number of jurisdictions that have been less affected because of preshortage reliance on IGRAs is unknown.

To address shortages of PPD TST antigen solutions, CDC has recommended any of three general approaches: 1) substitute IGRA blood tests for TSTs (2); 2) allocate TST supplies to priority indications, as determined by public health authorities (4); and 3) substitute Aplisol for Tubersol for skin testing, which depends on Aplisol availability (1).

Some surveillance programs for institutional or occupational TB infection control rely on routine serial TST or IGRA. For these indications, switching products or methods should be undertaken cautiously. Serial changes in test results become difficult to interpret, because the apparent conversions of results from negative to positive or reversions from positive to negative could be caused by inherent interproduct or intermethod variability (2,5). In settings with a low likelihood of *M. tuberculosis* exposure, the deferment of routine serial testing should be considered in consultation with public health and occupational health authorities.

Up-to-date information about shortages of biologic products, including PPD TST antigen solutions, is available from FDA online at http://www.fda.gov/biologicsbloodvaccines/safetyavailability/shortages/ucm351921.htm.

## Footnotes

* Additional information about TSTs available at http://www.cdc.gov/tb/publications/factsheets/testing/skintesting.htm.

## References

[1] CDC (2013). National shortage of purified-protein derivative tuberculin products. MMWR.

[2] CDC (2010). Updated guidelines for using interferon gamma release assays to detect *Mycobacterium tuberculosis* infection—United States, 2010. MMWR.

[3] CDC (2013). Reported tuberculosis in the United States, 2012.

[4] CDC (2000). Targeted tuberculin testing and treatment of latent tuberculosis infection. MMWR.

[5] CDC (2005). Guidelines for preventing the transmission of *Mycobacterium tuberculosis* in health-care settings, 2005. MMWR.

